# Supplementary material for: Are carbon emissions trading and green financial instruments synergistic? -Comprehensive quantitative research based on content analysis
Source: PLoS One. 2024 Mar 7;19(3):e0298601. doi: 10.1371/journal.pone.0298601 (PMC10919601; doi:10.1371/journal.pone.0298601)
Supplement: S1 Data — (ZIP) [file pone.0298601.s001.zip › Data/地级市Z_F工作报告.zip!/╣π╢1⁄2/╘╞╕í╩╨/└┤╫╘╣1⁄2╓┌║┼í╛┬φ┐╦╩2╛▌═°í┐_2008.docx]

各位代表：

我代表市人民政府向大会作政府工作报告，请予审议，并请政协各位委员和其他列席人员提出意见。

2007年工作回顾

2007年，市政府在省委、省政府和市委的正确领导下，在市人大及其常委会和市政协的监督、支持下，高举中国特色社会主义伟大旗帜，以邓小平理论和“三个代表”重要思想为指导，深入贯彻落实科学发展观，围绕市第四次党代会、四届人大一次会议提出的工作目标，在更高起点上继续实施“东渐西联、优内拓外、错位竞争、统筹并进”的发展战略，坚持走工业化、城镇化、农业产业化的发展路子，加快工业发展步伐，加大招商引资、项目建设、承接产业转移、发展县域镇域经济和民营经济工作力度，加快发展各项社会事业，着力解决民生问题，努力构建和谐云浮。经过全市人民的共同努力，圆满完成了市四届人大一次会议提出的经济社会发展主要预期目标。

2007年，全市实现生产总值290.77亿元，比上年（下同）增长13.7%，其中一、二、三产业增加值分别增长7%、20.9%和12%；单位生产总值能耗下降3.2%;全社会工业总产值373.02亿元，增长29.4%；农业总产值147.41亿元，增长7%；地方财政一般预算收入13.97亿元，增长22.7%；全社会固定资产投资139.56亿元，增长15%；社会消费品零售总额83.44亿元，增长19%；外贸进出口总额10.55亿美元，增长16%，其中出口总额6.75亿美元，增长19.8%；实际利用外资0.75亿美元，增长30.2%；农村居民人均纯收入4825元，增长7.4%；城镇居民人均可支配收入11658元，增长13%；居民消费价格总水平上涨3.6%;人口自然增长率5.56‰；城镇登记失业率2.37%。

——工业发展速度快、效益好，对全市经济发展的拉动力增强。积极帮助企业解决生产经营过程中遇到的水、电、油、运、土地、招工、资金等问题，引导企业增资扩产；运用高新技术和先进适用技术改造提升传统产业，推进企业做强做大；加大招商引资力度，加快工业园区建设，主动承接珠三角产业转移，促进工业加快发展。全市规模以上工业增加值74.29亿元，增长26.5%；利润总额增长61.4%，亏损面下降3%；综合经济效益指数146.5%，提高15.8个百分点。支柱产业快速发展，其中化工医药行业增加值增长47.1%、食品饮料行业增长34.7%、纺织服装行业增长30.5%、不锈钢制品行业增长28.5%、建材行业增长23.2%、电子行业增长23%，电力生产平稳增长。重大项目工作取得新进展。云硫集团年产40万吨硫酸扩建项目顺利动工建设；装机容量2×30万千瓦的云浮发电厂C厂项目列为省2008年动工建设的重点项目，国家发改委已经核准；云安县5万吨钛白粉项目获省批准建设；装机容量2×60万千瓦的华润西江发电厂项目列入省2008年重点建设前期预备项目，省已同意开展前期工作；4×100万千瓦云浮核电站项目进入可行性研究阶段，中广核集团已向省发改委上报项目建议书；云浮风电项目开展建塔测风等前期工作。承接产业转移工作取得了新成效。全市引进投资额500万元以上各类项目435个，计划投资总额224.5亿元，其中来自珠三角地区的产业转移项目183个，计划投资总额121.7亿元，项目涉及陶瓷、石材、房地产、纺织服装、化工、五金机电、旅游、农林产品深加工等行业。都杨和新成两个省级产业转移工业园累计投入建设资金2.28亿元，配套设施初步完善，已有部分项目入园发展；罗定、郁南分别与省内有关市（区）签订了共建产业转移园区协议，“广东省云安硫化工产业基地”已获省批准设立。

——农业和农村经济稳步发展，社会主义新农村建设扎实推进。克服持续干旱天气和猪蓝耳病疫情的影响，在确保完成粮食生产任务的基础上，加快发展农业产业化、特色农业和劳务经济，较好地实现了农业增产、农民增收、农村稳定目标。加大耕地保护力度，治理农田5.9万亩，新增耕地7700亩，连续8年实现耕地占补平衡。落实种粮直补政策，粮食作物播种面积237.58万亩，总产量96.5万吨，连续四年超额完成省下达的任务。农业产业化经营组织发展到165个，新增19个；实现销售收入150亿元，增长42.9%，带动农户24.5万户,户均增收4500元。温氏集团实现销售收入117亿元,增长53.9%。11个苦旱镇农业企业发展到23家，新增4家,带动农户2.8万户,户均增收2800元。优势农产品示范基地发展到50个，新增8个。完成4宗城乡水利防灾减灾工程和23宗小型水库除险加固工程。新增27个行政村通硬底化道路,提前两年实现全市村村通硬底化公路目标。新建生态文明村602条、沼气池4256座，农村生产生活条件不断改善。农村居民连续实现应保尽保，低保金总额增长31%；贫困户危房改造3319户，按期超额完成省定计划；农村住房实行政府出资统保；新型农村合作医疗基本实现全覆盖；农村中小学免收义务教育阶段书杂费政策全面落实。农村投资和消费两旺，固定资产投资、社会消费品零售总额分别增长22%和19%。农村外出务工人数、劳务总收入分别增长7.5%和8%，劳务经济成为农民增收的重要渠道。

——服务业发展加快，城乡消费市场畅旺。加大服务业招商和项目建设力度，大力推进商贸、旅游、房地产、物流等产业发展。消费品零售总额增长19%，其中批发贸易业、住宿餐饮业分别增长37%和57.9%。新建投资额500万元以上的第三产业项目38个，计划投资总额12亿元，建成24个。新增98家“万村千乡”省级农家店。开展创建中国优秀旅游城市活动，初步达到提高品位、推动产业、造福市民的创建目标。成功承办广东、广西10市旅游合作(云浮)联席会议，举办首届云浮旅游文化美食节，全年接待游客390.8万人次，实现旅游收入31亿元,分别增长27%和25.4%。新兴县被评为“广东省旅游强县”。全市房地产开发投资8.96亿元，增长47.5%。旅客周转量、货物周转量、港口货物吞吐量分别增长8.1%、25.4%和38.7%。通信、邮政、保险、证券、信贷、中介等现代服务业保持较快增长。

——县域、镇域和民营经济加快发展，经济总量不断壮大。充分发挥各地业缘、地缘和资源优势，坚持以工业化为龙头，加快农业产业化和城镇化发展步伐，以承接珠三角产业转移为重点，狠抓招商引资和项目建设，发展特色经济，促进了县域、镇域和民营经济加快发展。全市县域规模以上工业总产值230.76亿元，增长39.3%，其中云城、新兴分别增长52%和48.9%。各镇(街)新开工投资额100万元以上工业项目274个，投资总额57.5亿元。个体私营企业增加2778户，从业人员增长14.4%，注册资金增长8.4%，上缴税收增长19.1%，规模以上民营企业工业增加值增长42.8%。

——外经贸与招商引资工作成绩显著,外源型经济发展加快。加强外经贸和招商引资工作，积极参加第四届泛珠三角区域经贸合作洽谈会、第四届中国国际中小企业博览会、2007年粤港、粤台、广东—马来西亚经济技术贸易合作交流会等各种经贸活动，成功举办云浮（佛山）经贸合作洽谈会和第四届国际石材科技展览会，共签订500万元以上项目140个，计划投资额156.5亿元。其中云浮（佛山）经贸合作洽谈会签约项目48个，投资额91.5亿元。第四届国际石材科技展览会签订投资和贸易合同总金额22亿元，比第三届增长70%。驻珠三角招商联络处共引进合同项目105个，投资额5.64亿美元。积极应对国家出口退税政策变化，调整出口产品结构，加强出口通道建设，成立云浮检验检疫局，设立东骏、利仓行保税仓。新批自营进出口民营企业44家，新增出口额500万美元以上出口企业2家，对外贸易保持快速增长态势。

——财税金融工作平稳较快发展，经济稳健运行。坚持一手抓经济发展，培植税源；一手抓依法治税，强化征管。全市国税收入13.82亿元，增长20.9%；地税收入11.19亿元，增长16.8%。积极组织财政收入，科学安排财政支出。全市地方财政一般预算收入13.97亿元，增长22.7%，加上省税收返还、转移支付、各项补助以及上年结余等，实际完成财政总收入36.76亿元；全市完成财政总支出36.45亿元，其中一般预算支出35.97亿元，增长22.9%。坚持以人为本的公共财政理念，重点保障社会事业发展、新农村建设和解决民生问题的投入，全市城乡社区事务支出增长130.7%，医疗卫生支出增长34.7%，社会保障与就业支出增长30%，农林水事务支出增长28.6%，科学技术和教育等社会事业支出也有较大增长。金融服务经济社会发展力度加大。金融机构本外币存款余额281.98亿元，比年初增长11.9%；贷款余额116.71亿元，比年初增长10.2%。中小企业融资担保、地方金融资产追收、城市信用社退出市场、农村信用社呆帐清收和不良贷款压降等工作取得新进展。邮政储蓄银行云浮市分行挂牌成立，广东发展银行云浮支行正积极筹备开业。

——基础设施和生态建设成效显著，经济发展环境进一步改善。投入公路建设资金13.26亿元,完成公路建设改造613.5公里，超额完成年度计划。云城河口至郁南平台段高速公路全线动工，省道368线六都至罗旁段、云城河口至都骑码头公路改造建设基本完成。罗定至岑溪铁路、云浮港六都四围塘码头动工建设；南宁至广州高速铁路和都骑通用码头前期工作积极推进。新兴、郁南、罗定供电系统业务直管移交全面完成，全市用电同网同价；改造建设35千伏及以上输电线路49千米,在建110千伏及以上输变电工程6项。努力提高城市品位，优化人居环境，扎实推进城市重点项目建设，大力规范建筑市场秩序，推进建筑节能工作，强化建设工程安全质量管理。市区主要街道改造工程、河滨南路特色风情街、市迎宾馆三期建设基本完成；城区应急供水工程、市委党校新校区等建设继续推进。城区总体规划修编全面铺开，县城总体规划修编基本完成，镇、村规划工作走在全省的前列，郁南县大湾镇古民居村列为广东省第一批历史文化名镇名村，并报国家有关部门候选。严格执行建设项目环保准入制度，大力推进节能减排、城市环境综合整治、西江综合整治工作和治污保洁工程。重点在水泥、硫化工、石材等高耗能行业推广资源节约和综合利用技术，推行清洁生产，发展循环经济，取得良好经济、生态和社会效益。市区每月每平方公里降尘量4.59吨，连续多年优于省定标准；全市全年化学需氧量、二氧化硫排放量控制在省下达的年度目标以内。加大森林资源培育和保护，推进林业生态市、生态县建设，完成造林作业面积3.3万亩，纠正了一些地方毁林种果和违规种植速生桉现象，森林覆盖率达65.8%；罗定市、云城区分别成为“全国绿化模范县”、“广东省林业生态县”。建成全市电子政务信息平台和市、县、镇、村四级信息化服务中心，完善了全市信息化服务网络。

——扎实推进和谐云浮建设，各项民生工作取得显著成效。加强劳动和社会保障工作，新增就业岗位3.3万个,转移农村劳动力3万人；安排退役军人技能培训635人次；征缴社保费5.06亿元，增长17.4%。落实城乡最低生活保障、五保供养、城镇特困居民住房、水库移民后期扶持等工作，低保、五保发放总金额分别增长37.2%和30.7%；投入768万元建设廉租住房，解决城镇特困家庭的住房困难，市区困难家庭的住房问题得到妥善解决；投入2528.7万元改造建设23条水库移民村。农民工、妇女儿童、老年人、残疾人等群体的权益保障和农民减负工作进一步加强，全市4000多名儿童得到结对帮扶。产品质量和食品安全监管整治取得阶段性成效。严格落实安全生产责任制，安全生产四项控制指标全面下降，其中直接经济损失下降67.7%。抓好地质灾害防治，减少地质灾害造成的损失。落实人员、装备、经费动态保障机制，加强公安司法机关基层建设，全面完成镇（街）综治中心建设任务。加强社会治安综合治理，严厉打击各类违法犯罪活动，“两抢一盗”等恶性刑事犯罪得到有效遏制。设立市、县两级应急管理机构，有效提高处置公共安全和突发事件能力。加强人民调解和社会矛盾排查调处，落实信访条例和信访工作责任制，信访总量下降39.7%。

——扎实推进各项社会事业建设，社会文明程度不断提升。自主创新能力不断增强。新增省级以上科技进步创新载体16个，与高等学校、科研院所建立产学研合作关系的企业(协会) 20多家，我市被列为全省首批两个省部产学研结合示范市之一；温氏集团成为全省第二家组建大型企业研究院的企业；新增广东省著名商标4件，国家免检产品和国家地理标志核准注册各1个。教育事业健康协调发展。“一机两语”教育继续巩固和推进，我市被授予“广东省农村教育信息化示范市”称号；义务教育各类指标均达到省要求，高考上线率和录取率分别达77.3%和62.5%；中等职业技术教育招生增长10.5%；全面完成农村义务教育学校C、D级危房改造建设任务。文化广电新闻出版工作取得新成绩。建设了一批公益文化设施及博物馆、图书馆；举办了首届云浮民间艺术新春大巡游、石艺创意大赛以及全省性的《印象云浮》摄影大赛等文化活动；已有6个项目列入省非物质文化遗产保护名录，其中《禾楼舞》定为国家级名录公示项目。全面完成市直部门志编审。公共卫生工作进一步加强。我市成为全省首个农村中医工作先进市；改造薄弱卫生院10间，改善村卫生站829个，落实村医补助政策；新型农村合作医疗年补助上限提高到5万元，支付住院补助7.49万人次，金额9954.5万元。完善全民健身广场等群众体育设施，举办了市第三届运动会。全市人口出生率10.83‰，比省指标低0.77个千分点；出生人口性别比107.39，比上年下降0.55，较好地完成了省下达的人口与计划生育目标任务。土地管理责任制得到较好落实，矿产资源开发秩序进一步规范。武装、国防、人防、民兵预备役工作得到进一步加强，创建省双拥模范城工作扎实推进。物价、统计、人事人才、编制、知识产权、经济发展研究、民族宗教、档案、外事侨务、台务、消防、保密、气象、贸促会、工会、共青团、妇联、残联、侨联、工商联、文联、社科联、慈善会、红十字会以及驻市外机构等工作也取得新成绩。

——扎实推进民主法制和政府自身建设，政府管理效能明显提高。认真落实市人大及其常委会各项决议、决定并向其报告工作，主动加强与人民政协的联系并向其通报情况，办理人大代表建议111件、政协提案124件。密切与工会、共青团、妇联等人民团体的联系，广泛听取各民主党派、工商联、无党派人士的意见建议。不断完善村（居）民自治制度，不断推进政务、厂务、村务公开。在全省山区市率先开通了市、县、镇（街）三级行政服务网。在686个行政村推行“活力民主、阳光村务”工程，我市获得“全国村务公开民主管理制度创新奖”。在12个镇（街）开展建立社会群团组织试点。认真实施《行政许可法》、《公务员法》和《全面推进依法行政实施纲要》等法律法规，颁布实施规范性文件9件，办理审结行政复议案件30件，取消审批、审核、核准事项46项。进一步深化预算执行审计，部门预算和国库集中支付改革稳步推进。政府投资工程建设、经营性土地使用权和采矿权有偿出让以及国有、集体资产公开交易等制度进一步规范。政府采购预算金额2.7亿元,通过招投标采购节约资金10.7%。查处违纪违法案件、纠正损害群众利益的不正之风等工作取得较好成效。部门抓工作落实机制进一步完善，机关工作作风进一步好转，公务员队伍的大局意识、发展意识、机遇意识、责任意识和实干意识进一步提高，促进了各项工作的顺利开展。

各位代表！我市经济和社会发展取得的成绩，是在上级党委和政府的正确领导下，全市人民和社会各界人士奋力拼搏的结果。在此，我代表市人民政府，向全市人民、驻云浮部队官兵表示崇高的敬意！向关心支持云浮建设和发展的香港、澳门特别行政区同胞、台湾同胞和海外侨胞及国际友人表示衷心的感谢！

同时，我们也清醒地看到，我市经济和社会发展仍然存在一些较为突出的问题和薄弱环节。主要是：经济总量小，财力弱，县域经济发展不平衡；工业园区和基础设施建设与经济发展的要求仍不适应；农业基础设施薄弱，农民增收渠道不宽；教育及各项社会事业、民生工程投入以及历史债务化解压力仍较大。等等。对上述存在问题，我们要高度重视，并在今后的工作中创造条件切实加以解决。

2008年工作意见

各位代表， 今年是我市全面贯彻落实党的十七大精神,推动经济社会又好又快发展重要的一年。世界经济平稳发展，我国经济较快增长以及国际、国内特别是珠三角地区产业加快转移，为我市的发展提供了良好的外部环境；近年来我市基础设施逐步完善，投资发展环境日趋优化，招商引资和项目建设成效明显，全市干部群众求发展、抓发展要求强烈，经济发展后劲进一步增强。因此，做好2008年工作，市政府充满信心。只要我们解放思想，转变观念，勇于创新，大胆探索，抓住新一轮发展机遇，千方百计破除发展的瓶颈制约，加快完善推动科学发展的体制机制，坚持以人为本，关注民生，就一定能够促进经济社会的全面、协调和可持续发展。

2008年政府工作的总体要求是：高举中国特色社会主义伟大旗帜，深入贯彻落实科学发展观，认真落实党的十七大、十一届全国人大一次会议、省委十届二次全会、省十一届人大一次会议精神，按照市委四届三次全会的部署，以解放思想引领发展，以改革创新增创优势，以奋力拼搏寻求突破,以赶超争先提升形象，继续坚持走工业化、城镇化和农业产业化路子，紧紧扭住工业化这个龙头，不断加大招商引资和项目建设力度，优化经济结构，做大全市经济总量，增强经济实力；着力优化发展环境，加快改善经济发展环境条件；着力推进区域协调发展，促进全市经济加快发展；着力解决各项民生问题，让发展成果惠及广大人民群众；着力推进社会各项事业加快发展，促进经济社会协调发展；着力加强生态文明建设，打造我市生态优势，促进人与自然协调发展；着力推动干部队伍思想解放、观念转变和作风建设，促进干部队伍素质、工作效率和质量提高, 争当山区实践科学发展观与和谐社会建设的排头兵。

2008年经济社会发展主要预期目标是：生产总值增长13.5%，单位生产总值能耗降低3.5%，社会消费品零售总额增长18%，全社会固定资产投资增长15%，地方财政一般预算收入增长20%，外贸出口总额增长20%，实际利用外资增长30%，居民消费价格涨幅控制在4.8%以内，城镇登记失业率控制在3%以内，城镇居民人均可支配收入增长10%，农村居民人均纯收入增长7%，人口自然增长率控制在6.7‰以下。

实现今年经济社会发展预期目标,必须着重做好以下九方面工作：

（一）继续加快新型工业化发展步伐，不断推进全市产业结构优化升级。

充分发挥资源和产业优势，加大招商引资力度，积极承接产业转移，加快推进新型工业化进程，增强工业对经济发展的拉动力。加快发展工业产业集群。认真落实各项政策措施，强化产业发展规划,在做大做强石材、不锈钢制品两个省级产业集群的同时，积极引导硫化工、水泥、纺织服装、机械电子、生物制药、陶瓷及农林产品深加工等行业的企业加强生产、销售、技术合作与创新，发展新的产业集群；积极利用产业集群的集约经营优势，引进关联企业，壮大产业集群规模和产业配套能力；鼓励和扶持工业龙头企业进行技术改造，提高产能与效益；积极支持企业实施名牌带动战略，多创名牌产品和著名、驰名商标；积极筛选潜力好、竞争力强的支柱产业项目，与省联动共建产业结构优化升级工程，发展区域支柱产业。加快承接产业转移步伐。主动走出去，加强与佛山等珠三角地区各级政府、部门和行业商会的沟通联系与合作，举办多种形式的经贸合作洽谈会，及时掌握转移企业的信息，使产业转移工作取得更多更好的成效。今年将在佛山等珠三角地区举办两次产业转移经贸合作洽谈会。加快推进项目和园区建设。上半年动工建设云浮电厂C厂工程；华润西江发电厂项目争取尽快上报国家发改委核准，早日动工建设；加快推进省已批准的硫酸、水泥、钛白粉等重点项目建设；继续抓紧云浮核电站、云浮风电、粤泷电厂二期等大项目的论证申报工作。加快推进都杨、新成产业转移工业园配套设施建设，年内项目进园要有新突破；各县（市、区）其他工业园区也要加快规划、建设和项目引进工作。加快推进科技创新和省部产学研结合示范市建设。积极引进和推广科研成果改造纺织服装、石材等行业，重点组建和扶持不锈钢制品、石材、硫化工三大省部产学研战略联盟。加大科技投入和人才引进工作力度。年内争取实施省级以上重点科技项目19项，培育发展科技创新载体8个，科技进步对经济增长的贡献率达50%以上。加快推进民营经济发展。加强对民营企业的服务，大力培育民营龙头企业；推广温氏集团管理经验，促进民营企业建立和完善现代企业制度，提高管理水平；加强对民营企业主的培训，增强世界眼光、创新意识和品牌意识；组织企业开拓国内外市场。切实做好协调服务。完善重点项目建设台帐管理制度，加强对已签约项目的跟踪落实，加快动工项目的建设速度；加快信息化与工业化融合，以信息化促进工业化；想方设法帮助企业解决煤、电、油、运、用地、用工和资金等困难，保障企业正常生产；积极加强与上级主管部门的沟通联系，争取并落实相关扶持政策，推动工业快速发展。

（二）继续抓住发展县域、镇域经济这个重点，扎实推进社会主义新农村建设。

实现区域协调、城乡协调发展，建设社会主义新农村，关键在于加快县域、镇域经济发展。要按照“生产发展、生活宽裕、乡风文明、村容整洁、管理民主”的要求，在确保粮食播种面积保持在232万亩以上，粮食总产量稳定在87万吨以上的基础上，继续发挥各地业缘、地缘和资源优势，走工业化、城镇化、农业产业化发展路子，促进农村经济发展，加快新农村建设步伐。以发展县域、镇域工业为重点，壮大县域、镇域经济。继续实施县、镇两级经济工作责任考核制度；加快公共基础设施和产业转移园区建设，积极引进和发展资源加工型、劳动密集型和农林产品深加工型企业，大力发展具有特色和竞争力的产业。各中心镇要用足用活省、市扶持政策，加快经济发展，增强对周边村镇的带动能力；各专业镇要发挥现有产业的集群优势，做大做强特色产业；已规划建设工业园区的镇要进一步完善园区设施，引导企业入园发展；边远山区镇要在挖掘本地资源优势、积极引进项目的同时，用好异地办企业利益共享政策，实现加快发展。以农业产业化为龙头，促进农村经济发展和农民增收。充分运用“温氏模式与农业产业化发展高峰论坛”成果促进农业产业化发展。认真落实各项扶持措施，培育一批以种养业、农林产品加工流通为主营业务的龙头企业，大力发展生态农业和农林产品深加工业，提高产品附加值；实施市县两级单位挂钩指导和扶持石灰岩苦旱镇发展制度，增加苦旱地区农民收入。加强基础设施建设，改善农业生产条件。抓好基本农田和畜牧水产养殖业设施建设，年内改造中、低产田4.2万亩。抓好云城、新兴两个农业现代化功能区和全市50个优势农产品示范基地建设。切实加强对农业的服务。认真落实财政补贴和涉农税收规费减免优惠等政策。完善县、镇、村对农业的服务体系，大力推进农村信息化，提高对农业生产产前、产中、产后的服务水平。继续推进农业标准化生产，加强对重大动植物疫病防控和农产品生产环节的监管,保障农产品质量安全。加强农民知识技能培训，扩大农村富余劳动力转移就业规模，努力做大劳务经济。

（三）继续抓住发展现代服务业这个新增长点，加快壮大全市服务业规模。

服务业的发展程度是一个地区发达进步的重要标志。要抓住国家鼓励扶持服务业发展的有利时机，加快第三产业发展。加快发展商贸物流业。推进批发市场、专业市场和“万村千乡”市场工程建设，提高市场档次和管理水平；加强西江沿岸码头整治，加快西江航运开发，大力发展物流企业。加快发展旅游会展业。充分利用我市自然生态、人文历史、优质温泉等资源开发旅游项目，打造旅游品牌，发展特色旅游；加快发展餐饮酒店等行业；依托本地特色产业和名优产品，积极举办各类产业展销、技术博览、宣传推介活动。在积极参加国家和省举办的会展活动的同时，年内重点办好中国（云浮）新兴不锈钢餐厨具制品博览会和第五届中国（云浮）国际石材科技展览会、第二届旅游文化美食节。加快发展房地产业。以居民住宅小区建设为重点,发展满足不同消费群体需求的房地产市场。加快发展信息服务业。加强信息通信基础网络建设，加快政务、企业和社会领域信息化步伐。加快文化传媒、社区服务、中介服务、公共服务和科教服务等行业的发展，为促进生产发展、改善群众生活提供更好的服务。

（四）继续加大外经贸发展力度，进一步提高外经贸工作水平。

扩大对外经济发展空间。积极拓展国际市场，鼓励和支持民营企业申报自营出口经营权和免检产品。扩大纺织服装、不锈钢制品、石材等有自主品牌的产品和特色农林产品出口，大力培育新的出口企业与出口产品，努力扩大一般贸易占出口总额的比重。进一步加大招商引资工作力度。抢抓当前珠三角地区产业转移机遇，充分发挥政协港澳委员、海外社团组织和行业协会（商会）以及驻珠三角招商机构等的桥梁作用，参加并举办各种经贸活动，宣传推介我市产业、资源、区位、劳动力和园区优势，积极引进大企业尤其是高新技术企业到我市落户发展。鼓励外资企业增资扩产，做大做强。努力改善对外经贸发展条件。加快海关、检验检疫、港口建设，发挥石材和不锈钢制品保税仓作用，积极扩大生产设备、原材料、能源等物资和其它生产生活资料的进出口，做大对外经济贸易规模。

（五）继续加大财税金融工作力度，保障全市经济社会稳健运行。

坚持公共财政理念，加强财税金融工作，增强对经济社会发展的调节、保障和促进作用。努力提高财税收入总量和质量。加快二、三产业发展，培植壮大税源。坚持依法治税，协税护税，大力推进政府资源开发，规范非税收入管理，确保财税收入持续稳定较快增长。科学安排财政支出。深化财政管理体制改革，完善公共财政制度，优化支出结构，加大财政对新农村建设和科技、教育、文化、公共卫生、社保、社会治安、生态环境保护等的投入，让经济发展成果惠及广大人民群众。规范部门预算、政府采购、国库集中支付、财政支出绩效评价制度。加强国有资产管理，深化预算执行审计、效益审计和经济责任审计，提高财政资金和国有资产的使用效益。加快发展金融业。吸引国内外金融机构到我市设立分支机构,努力解决企业发展资金问题。切实加强金融监管，确保金融安全稳定。加快推进农村信用社改革，推动成立农村合作银行和统一法人联社。做好城市信用社退市工作。积极推动和支持优质企业上市融资，争取年内取得突破。支持保险、证券等机构发展新业务。

（六）更加注重基础设施和生态建设，不断改善经济发展环境。

加快交通基础设施建设。加快推进云城河口至郁南平台段高速公路、云浮港六都四围塘和都骑通用码头、罗定至岑溪段铁路建设及国道324线罗梅与罗冲段、省道368线罗旁至平台段、省道274线稔村至双阳段、河杨公路改造扩建，积极做好云浮至罗定高速公路前期工作，协助做好江门至罗定、阳江至罗定高速公路和南宁至广州高速铁路有关工作。年内全市交通建设计划总投入20.9亿元。加快能源基础设施建设。改造建设35千伏及以上输电线路196.3千米、110千伏及以上输变电站12座。年内全市电网建设计划总投入3.64亿元。加快城镇化建设。抓好中心城区重点工程建设，改造市区部分道路、横街里巷和城中村排水系统。推进市图书馆、博物馆、党校新校区教学楼、中级法院审判大楼、老干部活动中心建设，争取年内投入使用。强化建设工程质量安全管理，进一步规范建设工程招投标市场秩序，落实房地产市场调控政策。加强城市管理，优化人居环境。大力宣传实施《城乡规划法》，加强城乡规划修编和管理，抓好市城区总体规划、高速公路出口区域发展规划和县城、中心镇控制性详细规划修编工作。年内全市城镇建设计划总投入12亿元。加快水利基础设施建设。推进小型水库除险加固工程和城乡水利防灾减灾工程建设，确保按省的要求完成建设任务。继续开展农村饮水安全工程建设，争取解决8万群众饮水安全问题。年内全市水利建设计划总投入3亿元。加快生态文明建设。在工业、农业、消费等领域和企业、园区、城镇等层面大力发展循环经济。严格执行建设项目环境影响评价“三同时”制度，严厉查处违法排污行为，大力推进节能减排、污染源普查、大气污染防治、江河综合治理和治污保洁工程建设。大力开展清洁生产和资源综合利用，加快淘汰落后产能。加快污水处理厂等12个环保项目建设，年内建成6个。年内全市环保建设计划总投入2.4亿元。继续推进林业生态市和生态文明村建设，年内建设生态文明村600条、农村沼气池4000座。

（七）更加注重解决各项民生问题，努力推进和谐云浮建设。

努力促进社会充分就业。建立完善覆盖城乡的就业服务体系和就业援助制度，重点帮助下岗人员、零就业家庭、城乡低保户、退役军人、残疾人和被征地农民就业。年内力争新增就业岗位3.2万个，转移农村劳动力2.95万人。继续抓好农村青年、农民工等职业技能培训和智力扶贫、创业富民培训工程。年内计划培训2.52万人。认真贯彻《劳动合同法》，加强劳动合同管理，加大劳动争议处理和劳动保障监察工作力度，保障劳动者合法权益。完善城乡社会保障体系。以非公有制企业、城镇个体工商户和灵活就业人员为重点，大力推进社会保险扩面征缴。加强社会保险基金监管，确保社会保险待遇按时足额发放。全面开展城镇居民医疗保险试点工作。建立被征地农民社会保障制度，探索建立农村居民基本养老保险制度。努力解决早期离开国有企业和县以上集体所有制企业人员基本养老保险问题。健全城乡社会救助体系，完善农村“五保”供养机制。做好优抚安置工作。大力发展福利、慈善和残疾人事业。切实解决群众关心的热点、难点问题。采取发展生产、从严控制资源性产品和公共服务收费调价、建立价格变动监测预警制度以及依法打击物价违法违规行为等措施，努力防止价格总水平过快上涨。健全住房保障体系，扩大住房公积金覆盖面，加快经济适用房和廉租房建设,努力解决城镇低收入家庭住房难问题。继续抓好省人大水库移民议案工程建设，力争年内完成13条移民村改造建设任务。积极推进村村通客运工作。加强法律援助和司法救助，保障困难群众合法权益。积极解决农民工劳动权益保障、子女教育等问题。严格食品、药品等产品的市场准入和监管，构建保障产品质量的长效机制。切实抓好安全生产。严格落实各级政府安全生产监管责任和企业主体责任，切实排查和治理危险化学品、烟花爆竹、非煤矿山、道路交通、消防、建筑施工等重点行业和领域的安全隐患，坚决遏制重特大安全事故。扎实推进平安云浮建设。强化社会治安综合治理，进一步加大公安司法机关基层建设，坚持打、防、管、控并举，深入推进城市社区和农村治安防范工作，营造良好的治安环境。加强社会矛盾排查调处，切实解决群众信访反映的热点难点问题。完善应急预案和应急机制建设，提高应对突发事件的能力。

（八）更加注重发展各项社会事业，促进社会全面进步。

优先发展教育事业。落实城乡免费义务教育政策和预算内生均公用经费，加大高中阶段学校基础设施建设投入，增加学位，年内高中阶段毛入学率达到55%，比上年提高8.76个百分点。加快全市职业技术教育发展步伐，扩大地方高校招生规模。积极争取上级支持，年内立项兴建市级特殊教育学校。增强文化事业发展活力。加强基层文化设施建设，完善公共文化服务体系，全面推进农村电影放映、农家书屋、文化信息资源共享、镇级综合文化站和广播电视无线覆盖等工程建设。加强非物质文化遗产保护，努力打造南江文化、禅宗文化、石文化等特色文化品牌。加强文化市场执法和“扫黄打非”工作，规范文化市场秩序。加快医药卫生事业发展。深化卫生体制改革，推进农村三级卫生服务网络和社区卫生服务中心建设。落实镇卫生院、村卫生站财政补助政策。全面启动创建全国农村中医工作先进市工作。进一步巩固和完善新型农村合作医疗制度，提升服务质量和管理水平。完善疫病防控体系，加强卫生监督执法和药品监管体系建设。深入开展爱国卫生运动，抓好创建省级卫生城市工作。扎实做好体育、人口和计划生育、国土矿产资源管理等工作。进一步完善城乡公共体育设施建设，推进全民健身和竞技体育运动。抓好参加省第十三届运动会备战工作。创新人口和计划生育管理机制，稳定低生育水平，综合治理出生人口性别失衡问题。落实土地管理责任制，加强矿产资源管理和地质灾害防治工作。切实抓好应对气候变化的能力建设。做好创建省双拥模范城迎检工作。深入开展国家安全教育，做好国防动员、民兵预备役工作，抓好基层武装部建设。加强统计、人事人才、经济发展研究、民族宗教、外事、侨务、台务、妇儿、档案、方志、红十字会等工作。

（九）更加注重政府自身建设，努力提高行政管理和服务水平。

加强公务员队伍建设。以开展“继续解放思想，坚持改革开放，争当实践科学发展观的排头兵”学习讨论活动为切入点，增强加快发展的紧迫感，弘扬改革创新的开拓精神，清除制约科学发展的思想观念和体制障碍，强化公务员队伍大局意识、发展意识、机遇意识、责任意识和实干意识，努力建设政治坚定、业务精通、清正廉洁、作风优良的公务员队伍。加强民主法制建设。坚持和完善向人大及其常委会报告工作、向人民政协通报情况制度，自觉接受监督。认真办理人大代表议案、建议和政协提案。积极支持政协委员发挥参政议政作用。主动听取各民主党派、工商联、无党派人士的意见，支持工会、共青团、妇联、工商联等人民团体更好地发挥作用。积极开展法制宣传教育，深入贯彻落实《行政许可法》和《全面推进依法行政实施纲要》等法律法规，严格按法定权限和程序履行职责。巩固和扩大基层民主，深入推进 “活力民主、阳光村务”工程，做好村、社区“两委”换届选举工作。加强管理制度创新。在加强和改善经济调节、市场监管的同时，加快转变政府职能，努力创新和改进服务方式，积极探索社会组织承担政府转移或委托职能的实现形式，更好地为基层、企业和公众服务。进一步推动镇（街）社会群团组织工作，年内全市镇（街）建立社会群团组织的覆盖面扩大到60%。加快市、县、镇三级行政电子监察系统工程建设，扩大对政府服务的电子监察，规范行政执法，落实过错责任追究制，严肃查处各种失职渎职行为。继续精简和规范行政审批事项。健全部门工作协调机制，坚持分解年度工作要点、部门分工抓落实制度。完善电子政务建设，拓展网上受理、并联审批和“一站式”服务。继续通过行风评议、政风行风热线、市长热线、城市论坛等形式，扩大公众对政府工作的参与渠道。加强机关作风建设。树立优良政风，克服形式主义、官僚主义，把更多的精力放在抓落实上，主动为基层服务、为企业服务、为群众服务。建设节约型机关，坚持勤俭办一切事业。认真贯彻落实党风廉政建设有关规定，加强对权力的监督和制约，严防失职渎职、违法犯罪现象的发生。

各位代表！展望新的一年，市政府责任重大，使命光荣。让我们在党中央和省委、省政府的正确领导下，高举中国特色社会主义伟大旗帜，坚持以邓小平理论和“三个代表”重要思想为指导，全面贯彻落实科学发展观，解放思想，开拓创新，齐心协力，狠抓落实，为推进云浮科学发展与和谐社会建设作出新的更大的贡献！
